# Gharial nesting in a reservoir is limited by reduced river flow and by increased bank vegetation

**DOI:** 10.1038/s41598-021-84143-7

**Published:** 2021-02-26

**Authors:** Gaurav Vashistha, Ninad Avinash Mungi, Jeffrey W. Lang, Vivek Ranjan, Parag Madhukar Dhakate, Faiyaz Ahmad Khudsar, David Kothamasi

**Affiliations:** 1 Laboratory of Soil Biology and Microbial Ecology, Department of Environmental Studies, University of Delhi, Delhi, 110007 India; 2 Wildlife Institute of India, Dehradun, Uttarakhand 248001 India; 3 Gharial Ecology Project, Madras Crocodile Bank Trust, Mamallapuram, Tamil Nadu 603104 India; 4 Conservator of Forests, Western Circle, Haldwani, Uttarakhand 263139 India; 5 Biodiversity Parks Program, Centre for Environmental Management of Degraded Ecosystems, University of Delhi, Delhi, 110007 India

**Keywords:** Conservation biology, Population dynamics, Riparian ecology, Ecology

## Abstract

The gharial (*Gavialis gangeticus* Gmelin) is a fish-eating specialist crocodylian, endemic to south Asia, and critically endangered in its few remaining wild localities. A secondary gharial population resides in riverine-reservoir habitat adjacent to the Nepal border, within the Katerniaghat Wildlife Sanctuary (KWS), and nests along a 10 km riverbank of the Girwa River. A natural channel shift in the mainstream Karnali River (upstream in Nepal) has reduced seasonal flow in the Girwa stretch where gharials nest, coincident with a gradual loss of nest sites, which in turn was related to an overall shift to woody vegetation at these sites. To understand how these changes in riparian vegetation on riverbanks were related to gharial nesting, we sampled vegetation at these sites from 2017 to 2019, and derived an Enhanced Vegetation Index (EVI) from LANDSAT 8 satellite data to quantify riverside vegetation from 1988 through 2019. We found that sampled sites transitioned to woody cover, the number of nesting sites declined, and the number of nests were reduced by > 40%. At these sites, after the channel shift, woody vegetation replaced open sites that predominated prior to the channel shift. Our findings indicate that the lack of open riverbanks and the increase in woody vegetation at potential nesting sites threatens the reproductive success of the KWS gharial population. This population persists today in a regulated river ecosystem, and nests in an altered riparian habitat which appears to be increasingly unsuitable for the continued successful recruitment of breeding adults. This second-ranking, critically endangered remnant population may have incurred an "extinction debt" by living in a reservoir that will lead to its eventual extirpation.

## Introduction

Freshwater habitats occupy less than one percent of the Earth's surface, yet a tenth of all known species inhabit these areas, including a third of all vertebrate species^1^. Intense human pressures threaten the rich biodiversity in freshwater environments worldwide^2,3^, particularly the megafauna species which are at greater risk of declines and extinctions, than their smaller taxonomic counterparts^4^. The main threats include overexploitation, dam construction, habitat degradation, pollution and species invasion^5^. For example, dams not only interrupt river channel connectivity, but also have profound effects on riverine landscapes^6,7^. The major driver or grand structuring factor of river ecosystems is the natural flow regime, pulsating seasonally with floods and droughts^8–10^. River studies, firmly grounded in landscape-level perspectives^11,12^, are increasingly focused on dynamic models and management strategies that help predict restoration outcomes^13–16^.

For river-adapted habitat specialists, threats associated with loss of channel connectivity, altered flow regimes, and water extraction schemes are often direct and immediate and include increased harvest, restricted foraging opportunities, or loss of aquatic habitats. If the resultant impacts of these freshwater infrastructures, such as dams and irrigation canals, remain unaddressed, they can lead to species' reductions, fragmentations, local extirpations (e.g., Indus River dolphins^17^), and regional extinctions (e.g., gharial in the Indus^18^). Spatially, freshwater megafauna show the largest range contractions, approaching 99%, in the Indomalaya realm, higher than other regions^19^.

**Figure 1:**
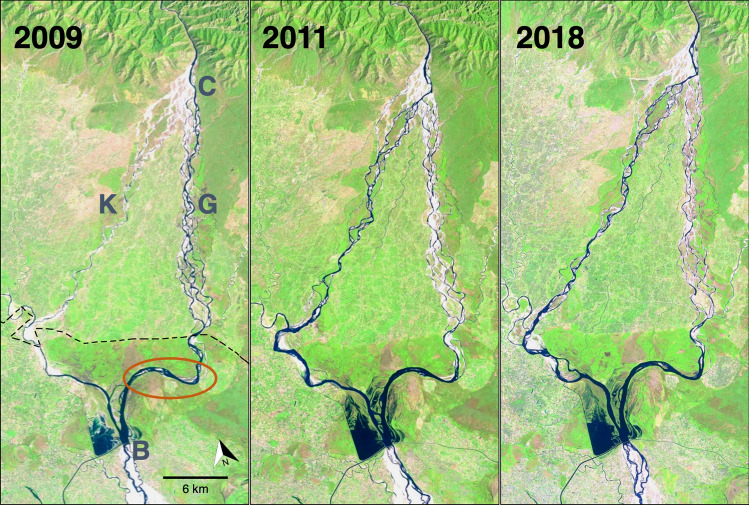
Satellite images before (2009; left panel) and after (2011 and 2018; middle and right panels) a flood event in 2010 in Karnali river channels, Nepal. The flood resulted in a mainstream channel shift from east (right side, = G, Geruwa channel in Nepal) to west (left side, = K, Karnali channel in Nepal). The Karnali mainstream naturally bifurcates below Chisapani (= C, above channel split) into two channels that flow into India across the international border (dashed line), forming the Kaudiyala (west branch) and Girwa (east branch) rivers. The reservoir created by the Girijapuri barrage (B = dam, barrage) maintained sufficient water level to support a breeding gharial population in a 10 km stretch of the Girwa (enclosed in orange oval). The barrage has control gates which allow water to flow downstream, but obstruct the movements of gharial upstream. Image source: Landsat series 4–5,7 (LandsatLook Natural Color Image, landsat 4–5 tm c1 level-1 and landsat 7 ETM + c1 level-1). Maps were generated from freely available satellite image data of U. S. Geological Survey (https://www.usgs.gov).

Natural flood events can result in channel shifts altering river discharge dynamics, and ultimately affect species composition, distribution and abundance. A recent example occurred within the Karnali River basin, the third largest riverine system originating in Nepal and flowing into India. In 2010, after monsoon floods, the active mainstream of the Karnali River shifted at a natural bifurcation from the east Geruwa (Girwa in India) channel to the west Karnali (Kaudiyala in India) channel (Fig. 1), resulting in attendant changes in river depth and flow from the east to the west channel. As a result of this natural channel shift, the relict Ganges river dolphin (*Platanista gangetica*) population shifted from east to west channel, but in doing so, moved from a protected stretch to one in which fishing and irrigation activities predominated^20,21^.

**Figure 2:**
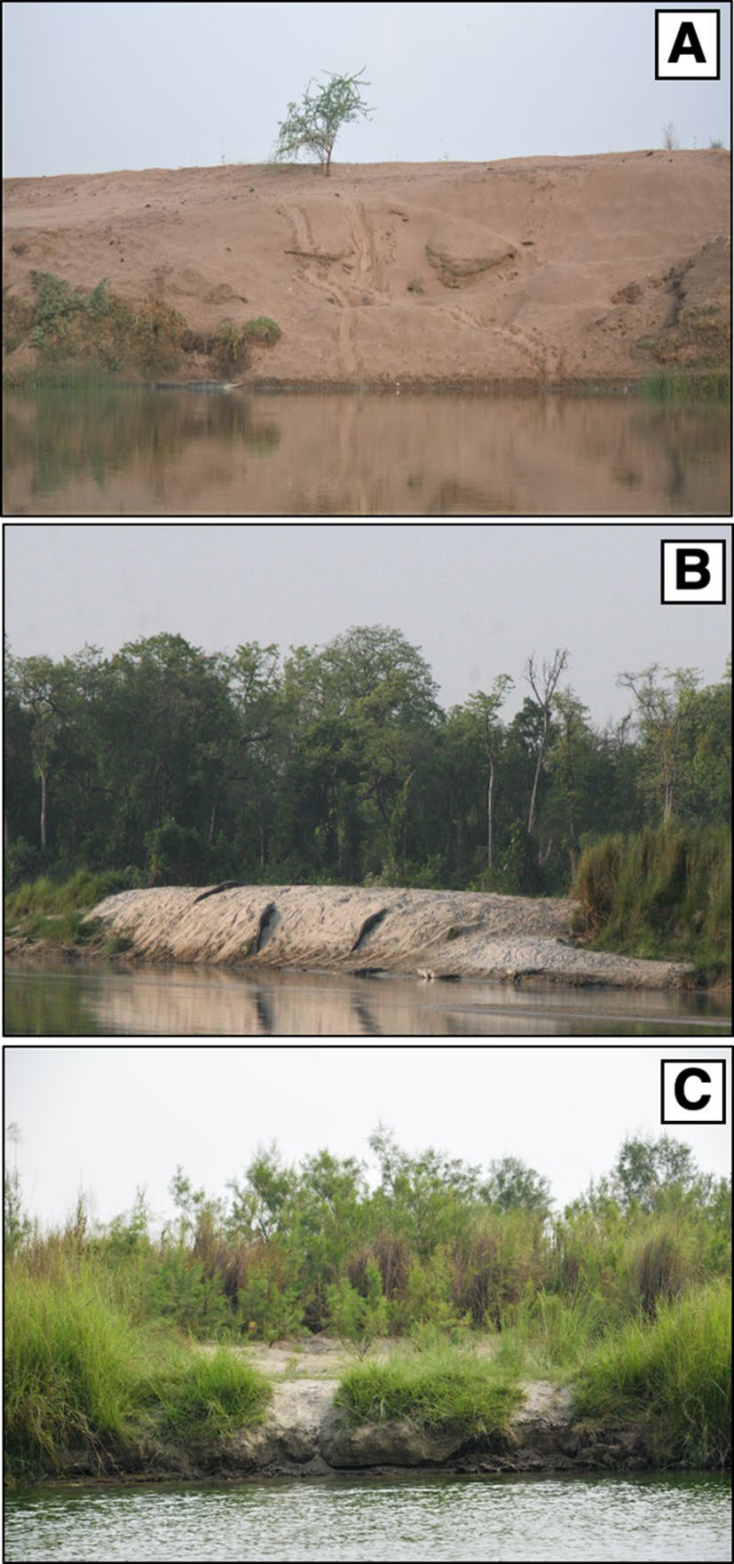
Nesting site of gharial in different habitats. **(A)** A gharial nesting site on Chambal river in National Chambal sanctuary, Madhya Pradesh. This site represents a typical sandy river bank which gharials naturally prefer for nesting. **(B)** A gharial nesting site in Girwa river in Katerniaghat before 2010 flood-channel shift. **(C)** Gharial nesting site N4 in Girwa river in 2018, post channel shift. (Image credits: **(A)**  Suyash Katdare; **(B)**  R. Whitaker).

Just downstream, across the Indo-Nepal border, gharial (*Gavialis gangeticus*) inhabits a protected stretch of Girwa River (20 km in length; = east branch of the Karnali River), within the Katerniaghat Wildlife Sanctuary (KWS). This small breeding population in the KWS is highly ranked globally, second only to the much larger Chambal population^18^. The species is a habitat specialist, formerly abundant in large rivers across south Asia. In particular, gharials nest on sand substrates such as mid-river sandbars and high sandbanks, adjacent to deep water pools^22^. The natural channel shift in the Karnali Basin in 2010, described above, has resulted in reduced river flow in the Girwa stretch where the gharials nest. As a consequence, there has been a marked increase in woody vegetation at previously utilized nesting sites, and a concomitant decrease in gharial nesting (Fig. 2C).

Decades of river studies have established that 1) the riparian environment is disturbance-driven, and 2) the main disturbing element is river flow fluctuation^23^. Floods continuously shape and rework river landscapes, and erosion and deposition create open banks and bars^24,25^. But, in regulated rivers, these forces are greatly reduced or absent^26,27^. To date, the most relevant studies dealing with processes and mechanisms have focused on downstream vegetation below dams^23,28–30^, but recent research aimed at understanding riparian vegetation dynamics in upstream deltas and backwaters of reservoirs is particularly informative^31,32^.

In this study, our goal is to investigate how the recent changes in riverine habitats are related to gharial nesting patterns in the Katerniaghat Wildlife Sanctuary (KWS), and how these features ultimately relate to natural and regulated flow regimes, within the Karnali Basin from Nepal into India. Here, we document (1) the long term trends and recent patterns of gharial nesting in the KWS, (2) the long term trends and recent changes in nesting habitats, and investigate, (3) how gharial nesting in the KWS may be limited proximally by an increase in woody vegetation, related to the recent channel shift, and ultimately by reservoir setting in a regulated river. Finally, we briefly discuss, (4) conservation strategies to maintain and enhance the KWS gharial population.

## Results

### Gharial population size, composition, and nesting (1975–2020)

Population counts of gharial and the number of nests located in the Katerniaghat Wildlife Sanctuary (KWS) on the Girwa River are summarized in Table 1, based on the available primary sources referenced. The number of reproductive adults increased from less than 10 in the late 1970s to close to 50 in recent years (2016–2020; Table 1). Notably, there was a concomitant increase in nesting, from less than 5 nests in the late 1970s to more than 30 in recent years (Table 1). Importantly, for most of the past four decades, although nesting increased, recruitment as indicated by the numbers of juvenile and/or yearlings in the smaller size classes, has shown little evidence of anticipated increases, based on either the observed increase in natural nesting and/or the numbers of captive-reared gharial added to the resident population via periodic releases of  > 1800 gharials (Table 1).

**Table 1:** Population counts and nest counts for the gharials resident in KWS, from 1975 to 2020.

Year	Adult males	Adults (< 3 m)	Sub-adults (2–3 m)	Juveniles (1–2 m)	Yearlings (< 1 m)	Total	Nest	Supplementation	Age of released Animals (in years)	Reference
1975–1980	1–3	8–9	4–7	7–12	7–8	14–34	3–5	178	3–5	46–48
1988–1991	2–4	2–4	14–24		3–4	36–39	10–15	0		49
1992–1995	3–5	3–5	10–20		3–7	39–54	15 +	301	> 10; 2–4	49
2000–2002	6	25 +	39 +		5 +	75 +	22–25	481	2–6	49
2004–2005	–	–	–		–	–	–	114	4–5	EFCCD
2006–2011	4–8	–	40 +		10 +	58–96	22–27	630	2–3	50–55
2016	8	38	11	0	9	66	35	0		PS
2017	7	40	8	3	7	65	32	0		PS
2018	–	–	–	–	–	–	25	116	3	PS
2019	7	44	8	5	0	64	19	0		PS
2020	6	40	17	9	0	72	36	32	4,7	PS

Ranges are shown, for available years (grouped by refs) for respective size/age classes, based on primary data (refs. cited). Supplementation refers to periodic releases of captive reared gharial, primarily juveniles and subadults; data provided by EFCCD, Uttar Pradesh.

*PS* present study.

### Gharial nesting site distribution (2015–2019)

Eight nest sites were confined to a 9 km stretch of the Girwa River within the KWS. Four sites were located on river banks (N1–3; N8) and the other four on mid-river sandbars (N4–7; Table 2, and Supplementary Data, S1 map and S2 natural history notes). In 2015, 5 of the 8 total sites had nests, increasing to 6 in 2016, and then to 7 in 2017. Then, the trend toward increasing nest sites reversed. In 2018, 3 sites were no longer used (N5, 7, 8), but a new site (N6) on an exposed mid-river sandbar was used. In 2019, only two sites (N1, N4) had nests.

**Table 2:** Nest site usage in Katerniaghat Wildlife Sanctuary by gharials, 2015–2019.

Location	Site no./type	2015	2016	2017	2018	2019
Pathrahna	N1/RB	+	+	3	5	13
Amba ghat	N2/RB	+	+	2	3	–
	N3/RB	0	0	2	1	–
Bhawanipur ghat	N4/SB	+	+	21	15	6
	N5/SB	+	+	1	–	–
Cement tower	N6/SB	0	0	0	1	–
	N7/SB	+	+	1	–	–
Madho nala	N8/RB	0	+	2	–	–
Total number of nests		NA	35	32	25	19

*RB* river bank, *SB* mid river sand bar, 0 = vacant site, +  = nesting (no counts), – = no subsequent nesting. *NA* not available.

From 2015 through 2019, the majority of nesting occurred at these two key sites, N1 and N4. Considering all 8 sites used during the five years, only these two sites had nests consistently year after year. Overall, N1 showed a tendency to flood, with reduced sand cover from 2017 through 2019 (N1, Fig. 5). N1 had the most nests in 2019, when nesting was restricted to only these two sites (N1 and N4), and was absent at the other sites (N2–3, N5–7; Table 2). A marked increase in woody cover (shrubs and trees) was evident at N4, coincident with a noted decrease in the number of nests, from 21 to 15 to 6, in 2017, 2018, and 2019, respectively (Table 2; Supplementary data, Figure S3).

### Habitat types based on vegetation cover

Vegetation at gharial nesting sites consisted of three grasses (*Phragmites karka, Saccharum spontaneum,* and *Typha* sp.), two herbs (*Euphorbia hirta* and *Ageratum conyzoides*), four shrubs (*Tamarix* sp., *Lantana camara*, *Ricinus communis* and *Calotropis giganticus*) and three tree species (*Adina cordifolia*, *Wrightia tinctoria* and *Bombax ceiba*). Open areas were mixtures of sand, water, and silt. There was an increase of 6% from non-woody to woody cover (*R*^2^ = 0.06 ± 0.04), but not in the sand cover (*R*^2^ =  − 0.45 ± 1). But otherwise, there were no substantial changes detected in the proportions of these categories at each site (Supplementary data, Figure S3).

### Habitat types demarcated using enhanced vegetation index

**Figure 3:**
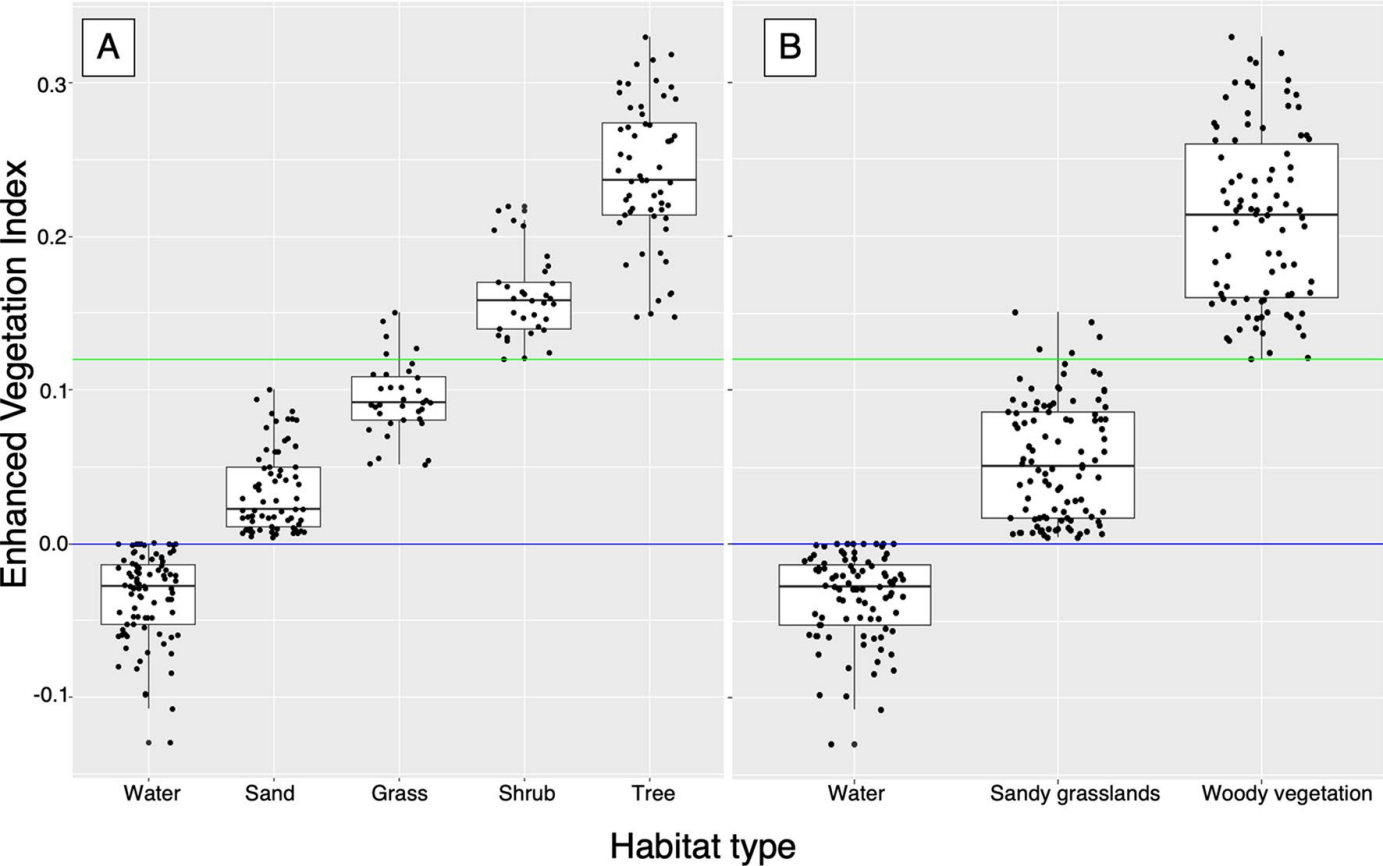
Assessment of the classification accuracy of EVI from 1988  to  2019. It revealed that water (EVI < 0, below the blue line) to have been classified most accurately. **(A)** Supervised classification using five habitat types. **(B)** Supervised classification after merging habitat types in three category. The sand and grass class was merged into one class of ‘sandy grasslands (0 < EVI < 0.13, in between blue and green line), while shrubs and trees were merged into ‘woody vegetation class (EVI > 0.13, above the green line).

An Enhanced Vegetation Index (EVI) was derived from high resolution Landsat 8 satellite images of vegetation patterns, as these related to the five habitat types associated with the observed gharial nest sites sampled in 2015 to 2019. The corresponding EVI values for the habitat types were: − 1 < water < 0; 0 < sand < 0.08; 0.05 < grass < 0.13; 0.13 < shrub < 0.215 and 0.215 < trees < 0.325 (Fig. 3A). When the above-derived EVI threshold were used to categorise the EVI from 1988  to  2019 into water, sand, grass, shrubs and trees, the average classifying accuracy across these classes was 66%. When the three combined types (water, sandy grasslands, and woody vegetation) were used to derive EVIs from 1988 to 2019, the overall accuracy of classifying increased to 94%, 91% and 88% for water, woody vegetation and sandy grasslands respectively (Fig. 3B).

### Habitat–nesting relationship (2017–2019)

**Figure 4:**
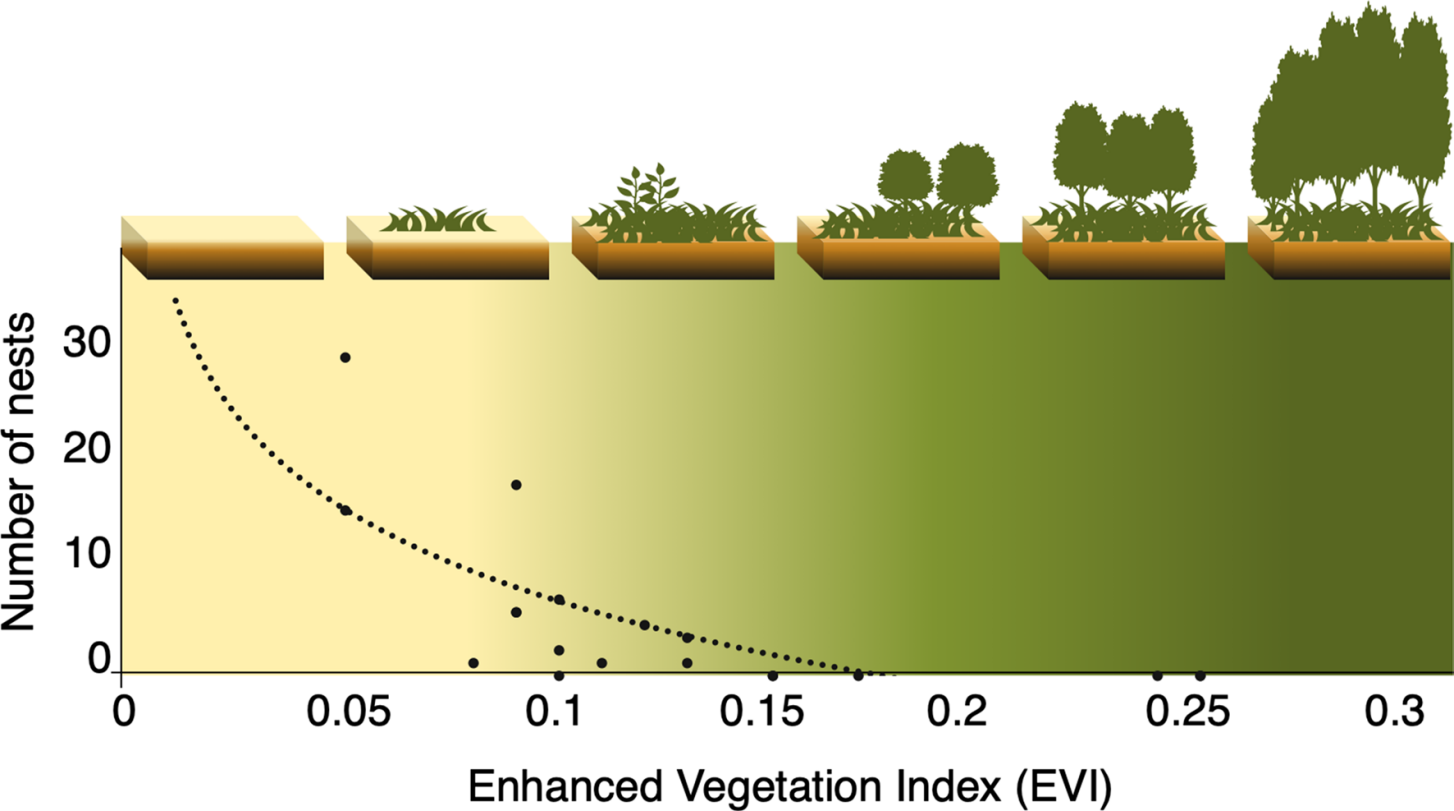
The relation between EVI and number of gharial nests in the study area. It revealed that nesting number decreased with increasing EVI. Gharial nests occur in a narrow habitat in between the river water (EVI < 0, brown shade) and woody vegetation (EVI > 0.13, green shade).

The high classification accuracy indicated above validated the long-term habitat classification of gharial nests. The relation between EVI and the number of gharial nests indicated a strong preference for sandy and grass habitat types, and a sharp decline in numbers of nests with increasing EVI, representing woody vegetation (*R*^2^ = 0.52, *p* = 0.0004) (Fig. 4).

### Long term habitat dynamics (1988−2019)

**Figure 5:**
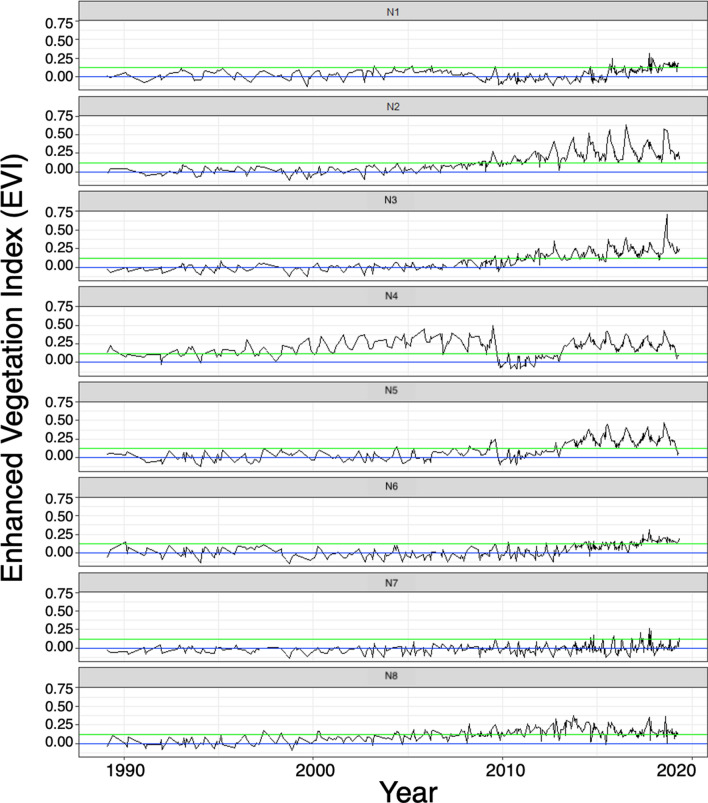
Habitat dynamics of gharial nesting sites from 1988 to 2019 in KWS. The EVI (*y-*axis) below the blue line represents water submerged habitat, between blue and green line represents mosaic of sand and grass (“sandy grasslands”), and above the green line represents mixture of shrubs and trees (“woody vegetation”). A habitat shift was observed across most of the sites around the year 2010, from which point the habitat gradually converted into woody cover.

Time-series analyses of the eight nesting habitats revealed long-term consistent seasonal fluctuations between water (EVI < 0) to sandy grasslands (0 < EVI < 0.13). These nesting habitats changed gradually or abruptly to woody cover (EVI > 0.13). The dynamics and shift point to the woody condition differed among sites. However, across all sites, a consistent change occurred with the flood-related channel shift in 2010. Specifically, sites were either converted to water followed by woody cover, or converted directly to woody cover (Fig. 5). Sites N2 and N3 had the highest shifts with a single prominent shift in 2010, after which a gradual shift from sandy grasslands to woody cover was observed. Site N4 changed from sandy grasslands to woody cover in 1998 and from woody cover to water in 2010, after which it gradually changed into sandy grasslands and in 2013 to woody cover until 2019. A similar pattern was observed at site N5, where it shifted from sandy grasslands to water in 2010 and gradually into woody cover from 2013 until 2019. In 2019, at both sites, there was a sudden shift back to sandy grasslands. This sudden shift from woody cover to sandy grasslands at N4 and N5 was due to an active intervention by the Forest Department to remove vegetation on these sites.

Site N1 had the least overall shift, with two prominent change points during 2010 (sandy grasslands to water) and in 2015 (water to sandy grasslands). Site N6 and N7 were mostly submerged in water until 2013, after which the variance expanded due to oscillations among water, sandy grasslands, and woody cover. At site N8, mostly sandy grasslands until 2008, there was a gradual shift to woody cover in subsequent years (Fig. 5).

## Discussion

Our study illustrates how reduced river flow, precipitated by a flood-related, natural channel shift adversely affected gharial nesting by promoting vegetation that impeded nesting at riverbank sites where nesting occurred in previous years. Specifically, from 2015 through 2019, the number of nesting sites declined by 70% and the number of nests were reduced by 46%. These decreases in nesting were coincident with a marked increase in woody vegetation at the established nesting sites used previously, related to the overall flow reduction in the east channel (Girwa = Geruwa), as the mainstream of the Karnali River upstream in Nepal shifted to the west channel (Kaudiyala). The observed effects were not immediate, but were observed subsequent to a major flood event in 2010. As a direct result of the flood, the Karnali River below the Chisapani Gorge shifted from the east channel (Geruwa), flowing into India as the Girwa, to the west channel of the Karnali, flowing into India as the Kaudiyala (Fig. 1). Prior to the flood event, the Geruwa had higher discharge and depth than the Karnali, and after the flood, the opposite was observed. In addition, subsequent to 2012, large scale water diversions from the Karnali west channel have occurred^20,21^.

In early 2011, within the Katerniaghat Wildlife Sanctuary (KWS), a 10 km stretch of the mainstream Girwa River, immediately below the Indo-Nepal border at Kothiaghat, still contained locally high densities of gharial, estimated at about 45 + adults^33^. Nesting at that time was estimated at about 25 + nests (Table 1). In KWS, the number of active nest sites reduced from 7 in 2017 to 2 in 2019. During the study period, 6 out of 8 nesting sites were dominated by native and invasive woody species (e.g., *Lantana camara*), making them inaccessible for nesting. As a consequence, the number of gharial nests that were detected also declined in 2016 from 35 to 19 in 2019 (Table 1).

Our analysis of the vegetation changes at known nesting sites, by monitoring the dynamics between water, sandy grasslands and dense woody vegetation on the ground as well as via remote sensing, revealed that these habitats had fluctuated along the river banks in response to high water pulses from periodic floods. The riverine landscape experienced major flood events around 1995, 2000, 2008 and 2010^34^. After the 1995 and 2000 floods, most of these sites gradually changed into sandy grasslands and dense vegetation, until they were submerged by another extreme flood in 2008 that removed the dense vegetation. However, these vegetation changes ceased after the 2010 flood. In subsequent years, the sampled areas were eventually converted into densely packed woody vegetation, without the periodic reversals to water, sand or grass stage. With exception of one site predominantly covered by grass (N7), all of the other gharial nesting sites were covered by woody vegetation (Figs. 2C, 5).

However, discussing habitat dynamics using moderate resolution data is challenging, so additional documentation of the shift to woody vegetation would be instructive. For instance, a single pixel of satellite imagery may constitute more than 75% water and 25% sandy grassland, but because of the classification method it is classified as water. In another scenario, a pixel might constitute > 90% vegetation and is classified as woody. In both of these settings, classified as either "water" or "woody," if small patches of sand were present, these may have been suitable sites for nesting gharial, but such patches would have not been detected at the resolution level used in this study.

Therefore, our estimate of nesting habitat and its dynamics require refinement with high resolution satellite data. Moreover, for a large-ranging aquatic species known to utilize long stretches of free flowing rivers^35,36^, our study area was relatively small (< 20 km). A small sampling area with just eight nest sites, precludes general comments about floodplain vegetation dynamics in an unregulated river system. Lastly, due to the absence of adequate hydrological data for the east (Girwa) vs. west (Kaudiyala) channels, a direct assessment of the relative channel discharges following the 2010 flood could not be done. However, upstream just across the border in Nepal, a small relictual population of Ganges river dolphins shifted from the east channel (Geruwa) to the west channel after the 2010 flood, coincident with documented depth changes in the channels, from deep to shallow in the east channel, and vice versa in the west channel^20^. There were reductions in river depths in both channels after 2012 as a result of an increased irrigation demand upstream near the branch point at Chisapani^20^, as well as increased water extraction from east channel to west channel within the braided inter-channel region of the Karnali Basin^37^.

In other localities, gharial prefer to nest on high sandy banks, devoid of vegetation and adjacent to deep water, e.g. Chambal River nesting sites^35,36^ (Fig. 2A). In comparison with Chambal River sites, the nesting sites described here for KWS and characterized by fluctuating and higher EVI values (EVI > 0.13) present a completely different scenario, related to the altered riverine ecosystem associated with overall reduced river flow in the reservoir landscape. Habitats such as sand and grass (EVI < 0.13) provide easy access to such localities, compared to sites with woody vegetation (EVI > 0.13) (Fig. 2B). In this setting, the high water table related to the presence of the Girijapuri barrage and resultant reservoir generally promotes the growth and proliferation of riverside vegetation, particularly in the broad deltaic areas where inlet channels with reduced flow join the reservoir^31,32^. In the KWS, the recent reduction in river flow in the Girwa River related to the channel shift away from the mainstream Karnali has likely contributed to the loss of potentially suitable nesting sites in several additional ways. Included here is the loss of scouring action by floodwaters that would periodically clear riverbanks of vegetation, as well as the lack of "new" sediment deposition sites created by floodwaters, such as sand and/or gravel banks or bars that would create nesting sites de novo^23,29,30^.

Nest site selection is an important factor that contributes to successful breeding in reptiles^38–41^. Vegetation has several direct and indirect effects on the nesting of crocodylians. It can reduce accessibility to potential nesting area, influence hatchling sex ratio, increase embryo mortality and change physical parameters of the substrate^42^. Our observations indicate that the invasion of woody vegetation limits a gharial’s access to potential sites in the KWS, making these overgrown sites unfit for nesting (Fig. 2C). It can further affect maternal care by restricting the female gharial’s access to nests, resulting in death of fully grown hatchlings. During our sampling period, we found evidence of physical damage to shells of incubating eggs by vegetation roots that ultimately resulted in egg mortality. In addition, in gharial, incubation temperature determines hatchling survival as well as many important attributes, so suitable nesting substrate and other physical features of the nesting site play important roles in nest success^43–45^.

In the KWS, the small remnant gharial population is vulnerable to limited habitat size, lack of suitable nesting sites, hatchling predation (including egg poaching by local people) and poor hatchling recruitment. Status reports, primarily winter basking counts of adults and nest numbers, provide snapshots of population numbers and composition^46–55^ (Table 1). The total population size and nest numbers have gradually increased since 1975, but the increase is almost negligible compared to numbers of captive-reared, released gharial added periodically to the KWS population. Restocking with captive reared "head-started" gharial, primarily juveniles, was initiated in 1979, and has continued through 2020, with the cumulative releases totaling 1852 to date (Table 1). But in reality, very few of the head-started animals released in KWS actually became residents there, as is clearly evident from low numbers of juvenile gharial in the population counts (Table 1).

The plausible reason for a chronic failure to recruit young animals is that most, if not all, are simply moving downstream below the reservoir dam, and are not able to move back upstream, once they cross the Girijapuri barrage gates and enter the Ghaghara River. This situation applies to young individuals, both wild hatched residents as well as captive-reared releases. The loss of nesting habitat for the gharial residents in the Girwa river, that we have documented in this study, could result in local extinction of this species in the KWS. If this gharial population were not able to recruit successfully, as it appears may now be the case, its loss may well qualify as the unfortunate payment of an "extinction debt," e.g. a delayed species extinction as a consequence of an ecosystem perturbation^56,57^, directly related to the dam and reservoir established in 1976. Poor recruitment despite the addition of hundreds of supplemented individuals is a serious problem confronting the persistence of this long-standing reservoir population.

Survival of the KWS gharial population may well depend on active and immediate intervention to reverse multiple factors contributing to this obvious lack of recruitment. Two management interventions were piloted at the KWS nesting sites recently: (1) vegetation removal by clearing vegetation on nesting banks, and (2) sand addition by shifting sand to extend river banks. One effective immediate solution could be providing artificial sandbanks by simply piling sand at the river's edge to simulate naturally occurring sandbanks that formed at KWS prior to the channel shift post-flood. This has proved remarkably successful in 2020 at KWS. Newly constructed sites where sand was added were readily utilized for nesting, resulting in nearly double the number of nests (Vashistha et al. 2021, Unpublished data; also see Table 1).

The long term solution will necessitate retention of juveniles within the KWS system, and their eventual recruitment into the adult breeding population. Unfortunately, there is little advantage for the resident gharials in the KWS to shift westward into the unprotected mainstream Kaudiyala channel where the river flow conditions might periodically fluctuate sufficiently to create suitable nesting habitat, because the west channel is intensively cultivated and fished^20,21^. Upstream in the Karnali Basin, below Chisapani, gharial have largely been eliminated in recent years, with little to no evidence of breeding and only the occasional animal sighted between the Indo-Nepal border and Chisapani, in either channel within the past several decades^58^. Movement upstream in the east channel across the Indo-Nepal border from KWS into the Geruwa is less likely due to the reduced flow since the 2010 flood, and the 7 km stretch of the Geruwa upstream from Kothiaghat is unprotected below the Bardiya National Park boundary. Our results suggest that one possible solution may involve a cooperative initiative by the two countries in the Karnali basin—India and Nepal—to develop a collaborative conservation agenda for this important riverine landscape while it is still largely intact and free-flowing^59^.

## Methods

### Study site

Our study focused on 20 km of the Girwa river from Girijapuri barrage (28°16′21" N 81°05′13" E) to Indo-Nepal Border (28°22′02" N 81°12′05" E) in Katerniaghat Wildlife Sanctuary (KWS). This sanctuary has an actively breeding gharial population with 47 adults^18^. This is the only breeding area remaining for gharial in the Karnali basin^18^. The study site consists of extensive alluvial plains, hygrophilous grasslands and tropical moist deciduous forests. Girwa channel width was 403.18 ± 150.25 m during 2017–2019 and the river is interspersed with sand bars. Owing to freshwater demands for agriculture and human consumption along the river banks, infrastructures such as a barrage (Girijapuri barrage) and irrigation canals (Sharda Sahayak link canal and Saryu Nahar Irrigation) were built at the confluence of Girwa and Kaudiyala inside the KWS. The barrage marks the downstream boundary of KWS gharial population. It is opened at least twice each year, once for gate maintenance in April and again during the summer monsoon. The Girijapuri barrage presents a one way travel route for any gharials moving downstream, especially hatchlings and juveniles. Upstream movement across the barrage is not possible when the gates are closed. Hence the barrage likely plays a key role in the population dynamics of the gharials upstream in the KWS, but movement data to support this supposition is lacking.

### Gharial population data

We assembled available data on gharial population counts and on nest counts from primary sources, published as well as unpublished (Table 1). Population counts for 2016–20 were conducted by boat surveys in January–February annually. The surveys started in Girwa river from Pathrahna, went downstream till Girijapuri barrage and then upstream in Kaudiyala river to the Indo-Nepal border. The same observers did all the population counts. Gharials were recorded based on different size classes (see Table 1). The Environment, Forest and Climate Change Department, Uttar Pradesh (EFCCD) provided access to records on supplements to the resident KWS gharial population, periodically released at various locations along the Girwa River in the reservoir.

### Gharial nesting site distribution

Historical records of nesting indicate that nesting from 1975 to 2015 when our study started, was confined to the same 10 km stretch of the KWS that we identify here (Table 1). During the years 2017–2019, this area where gharial nested previously was surveyed in late March and early April to locate trial nests, and completed nests, using distinctive and prominent identification cues such as open holes with spoor marks (Supplementary data, figure S4). Information pertaining to date, time, habitat cover and distance to water for all completed nests were recorded, as well as GPS coordinates. We identified eight sites (N1–N8). The EFCCD staff at KWS provided nest data for 2015–2016.

### Habitat plots on nesting sites

Between 2017 and 2019, the areas of nesting sites were measured, and six 1 m^2^ plots were marked at each site. Habitat plots were located on sloped riverbanks and flat mid-river sandbars. These plots were used for ground truthing the habitat type for each site based on vegetation cover and enhanced vegetation index (EVI). For analyses using vegetation cover, three habitat types were considered, consisting of "herbs", "woody" (shrubs and trees), and "sand" (sand and silt).

For EVI based classification, five habitat types (water, sand, grass, shrub, tree) were identified for this study. Water habitat consisted of plot submerged under water for at least 5 cm depth. Sand habitats were barren areas covered with sand and silt and lacking vegetation. Grass, shrub and tree habitats were areas that were covered by monocotyledon plants (excluding bamboo) or woody dicotyledon plants taller than 30 cm or woody dicotyledon plants taller than 200 cm respectively. Habitat plots were classified into different habitat types based on criteria mentioned in Table 3. The differential threshold used to determine the plot dominance was based on the relative contribution of different habitat types to the plot cover. For instance, even if a single tree was present in a plot (i.e. cover > 10%), it hinders the growth of grasses under it; while a relative dominance by grasses was possible only when more than a third of the plot (i.e. cover > 30%) was covered with grasses.

**Table 3:** Habitat groupings, based on surface features measured in a habitat plot.

SL. No	Habitat type	Criteria used for assigning an individual plot to a habitat type
1	Water	> 75% of the plot’s surface was covered by water and the rest of the plot was either sand or grass
2	Sand	> 75% of the plots surface was barren sand and the rest of the plot was water or grass
3	Grass	> 30% of the plots surface was covered by grass and the rest of the plot was water or sand
4	Shrub	> 10% of the plots surface was covered by shrubs (woody dicotyledon plants taller than 30 cm) and the rest of the plot was water, grass or sand
5	Tree	> 10% of the plots surface was covered by trees (woody dicotyledon plants taller than 200 cm) and the rest of the plot was water, grass, shrub or sand

### Habitat types classification using enhanced vegetation index (EVI)

For understanding the habitat changes and its relation with the study species, it was important to correlate the habitat with satellite remote sensing data so as to monitor long-term changes. In order to establish a relationship between the sampled habitat plots and satellite data, we used high resolution Landsat 8 satellite images for the sampled locations to derive an Enhanced Vegetation Index (EVI) following Jensen^60^.

The EVI for all images captured in March and April of every year were averaged to obtain an average EVI for every habitat plot for every year for the period 2015–2019. A random subset of 80% habitat plots was used to assign an average EVI value. This assignment provided the data variability in EVI values for the five habitat types. This data was used to classify the averaged EVI into these five habitat types using supervised classification in ERDAS Imagine Software (Version 2015). The remaining 20% habitat plots were used to validate the parity between habitat types recorded on ground to the habitat types mapped derived from EVI, using Kappa statistic^61^. Due to overlap in signature values of sand and grass, as well as shrubs and trees, we combined these five habitat types resulting in only three habitat types, i.e. water, sandy grasslands and woody vegetation.

### Long-term habitat dynamics

Landsat EVI effectively captures long-term changes in vegetation cover at an optimal resolution. To estimate vegetation cover trends for the last three decades, EVI acquired from Landsat series 4, 5, 6, 7 and 8 for the years 1988***–***2019 was used. For each sampled location, EVI values were compiled for all available scenarios across different months over a period of 31 years. A total 357 scenes of different time periods were used for each site. Using the aforementioned thresholds of classifying the EVI into five habitat types, the EVI values were classified into the five types for each nesting site. This provided the first time series product of the habitat types in this landscape. However, across the considered 31 years, Landsat satellite sensors and resolution have changed substantially. This may affect EVI accuracy and band reflectances. To address this issue, we visually validated a subset of EVI classified scenes for every year by visually comparing the classified habitat map with Google earth imagery. First we trained our visual interpretation of the Google earth imagery by using the images of 2018, during which we sampled the habitat and compared the imagery with sampled habitat plots and associated EVI values. Subsequently, we assessed at least one Google earth scene per sampled location per year to record the type of habitat. In total, there were 88, 67, 35, 33 and 57 observations for water, sand, grasses, shrubs and trees respectively. We matched this visually interpreted habitat type of each nesting site with the categories derived from the closest classified EVI. Parity was assessed using Kappa statistic.

Post validation, the habitat categories were compared across the temporal scale from 1988 to 2019 to visualize the seasonality and shifts in the habitat dynamics of each nesting site. We used this time series model of the habitats to identify timeline points after which a shift was observed in mean and/or variance of the habitat dynamics, using the R package CHANGEPOINT^62^. The point, following which there was no return to prior dynamics of the habitat, was considered as a point signifying potential regime shift^63–65^.

### Gharial nesting and riverine habitat changes in KWS

We looked at the changes in the gharial nesting site distribution from 2015 to 2019 from information collected during nesting surveys and secondary data. Active and inactive nesting sites were identified for each year based on nesting efforts to determine the change in habitat use for nesting. We further analysed the relation between the habitat dynamics and gharial nesting based on the visual observations of the habitat dynamics and potential regime shift. EVI, a derivative of habitat type and satellite imagery assessment, was compared with the nesting effort. The average EVI of the nesting season was compared with the number of actual nests. Trial nests were not included in the assessment. For comparison, we assessed the fit of linear, exponential and logarithmic lines. The line with highest *R*-squared value was considered as the best representation of the relationship between gharial nest number and the EVI. Precision of the fit was assessed by 1000 bootstrap replications in R version 3.3.1^66^, wherein we selected 80% of observation points each time, assessed the *p*-value and reported the average *p*-value.

### Ethics statement

This study was based on non-invasive sampling such as boat surveys, vegetation sampling and satellite imaging. No animal handling was involved and therefore the study was not assessed by an animal ethics committee. All necessary permissions for entry into protected areas were obtained from the Environment, Forest and Climate Change Department, Uttar Pradesh (EFCCD), vide letter no. 2522/23-2-12(G) dated 31st March 2016.

## Supplementary Information

Supplementary Information.
