# Supplementary material for: Gharial nesting in a reservoir is limited by reduced river flow and by increased bank vegetation
Source: Sci Rep. 2021 Feb 26;11:4805. doi: 10.1038/s41598-021-84143-7 (PMC7910305; doi:10.1038/s41598-021-84143-7)
Supplement: Supplementary file 1 — Supplementary Information. [file 41598_2021_84143_MOESM1_ESM.docx]

Gharial nesting in a reservoir is limited by reduced river flow and by increased bank vegetation

Gaurav Vashistha, Ninad Avinash Mungi, Jeffery W. Lang, Vivek Ranjan, Parag Madhukar Dhakate, Faiyaz Ahmad Khudsar, David Kothamasi*

* Corresponding author: Dr. David Kothamasi

Laboratory of Soil Biology and Microbial Ecology, Department of Environmental studies, University of Delhi, Delhi 110007, India

Email: [dkothamasi@es.du.ac.in](mailto:kothamasi@hotmail.com)

**Supplementary Information:**


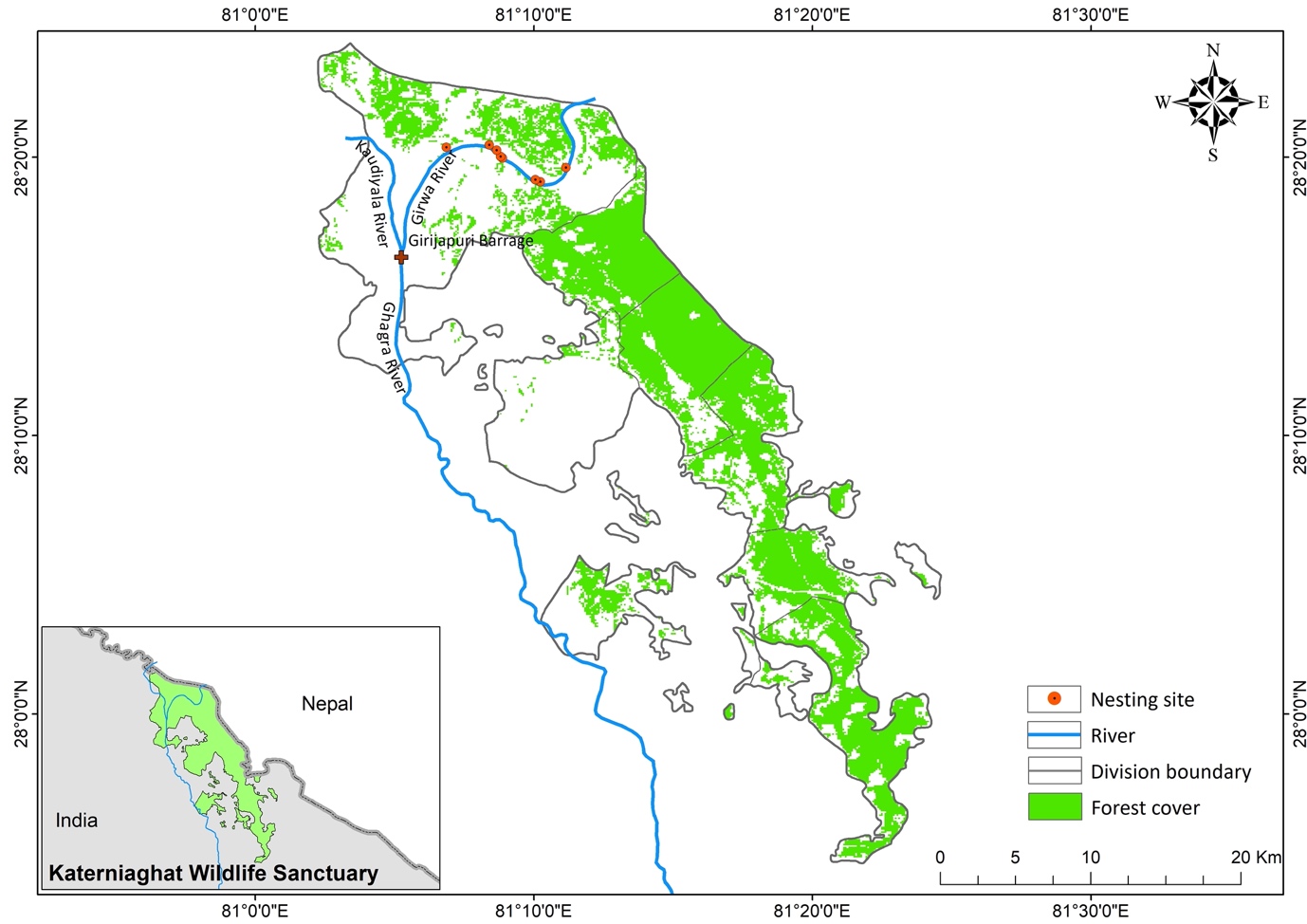


Figure S1: Location of nesting sites of gharial at Katerniaghat wildlife sanctuary (2015–19). Map was generated using ArcGIS 10.3.1.

S2: Natural history account of gharial nesting sites in the study area

| Site | Site Description |
| --- | --- |
| N1 | Situated at Pathrahna was a mid-river sandbar until 2009–10. There was presence of vegetation, probably grass species. By 2011–12, an exposed river bed connected the sand bar to the forested area. In 2014 the sand bar itself was completely covered by vegetation consisting primarily of *Phragmites karka*. The connecting barren river bed was succeeded into grassland by 2018 while the sand bar was succeeded by mixed vegetation of both grass and woody tree species. The slope on the edge of river bank at this site is still in use for gharial nesting. Gharial females have attempted trial nesting on the nearby sand bars, however no nests were found there |
| N2 and N3 | Situated at Amba Ghat were a mid-river sand bar since before 1996 till 2008–09. The river bed in western channel gradually accumulated sediment and was exposed to vegetation succession. The completely barren channel was covered with vegetation in less than two years (2011–12) and the mid river sandbar got connected to the forested area. The sites were being used for nesting till 2018 but sediment accumulation near the site (80 m wide) has rendered it unsuitable for gharial nesting. |
| N4 – N7 | Situated at Bhawanipur Ghat were located on mid river sand bars which were present since before 1996. These sand bars were a continuous stretch (~2000 m long) which kept changing its dimensions each year. The sand bars were barren in 2009–10 and first sign of vegetation were seen in 2011–12, at the present day N4-N5 site location. A vegetation patch of 4.5 hectare was recorded in 2014. The continuous sand bar got divided into two patches in 2015–16. The bigger sand bar patch has sites N4 to N6 and the smaller sand bar patch has site N7. The smaller patch was 2.21 hectares in 2017 and was completely covered with vegetation, dominated by *Saccharum spontaneum* and *Phragmites karka*. The bigger sandbar 25.49 hectares in area, of which 14.91 hectares (58.49%) was covered under vegetation, which increased to 23.90 hectares (93.76 %) in 2018. Only 2.83 hectares (11.84 %) area is covered under mix vegetation consisting of both grass and woody tree species. Dominant species were *Adina Cordifolia*, *Euphorbia hirta*, *Wrightia tinctoria*, *Tamarix* sp. and *Bombax ceiba*. The remaining 20.07 hectare (89.28 %) was under grass cover dominated by *Saccharum spontaneum*. |
| N8 | located on Madho Nala. Madho nala flows from the main river, inland into the forest area to join a bigger drainage into Kaudiyala River. Madho nala was well connected to the main river and functional till 2014–15. In the year 2016–17, its mouth at Girwa river was blocked by sedimentation and connectivity with the main river was cut. In the following year the recently blocked mouth (of Madho Nala) was covered initially by *Saccharum spontaneum* and succeeded by forest woody species. |


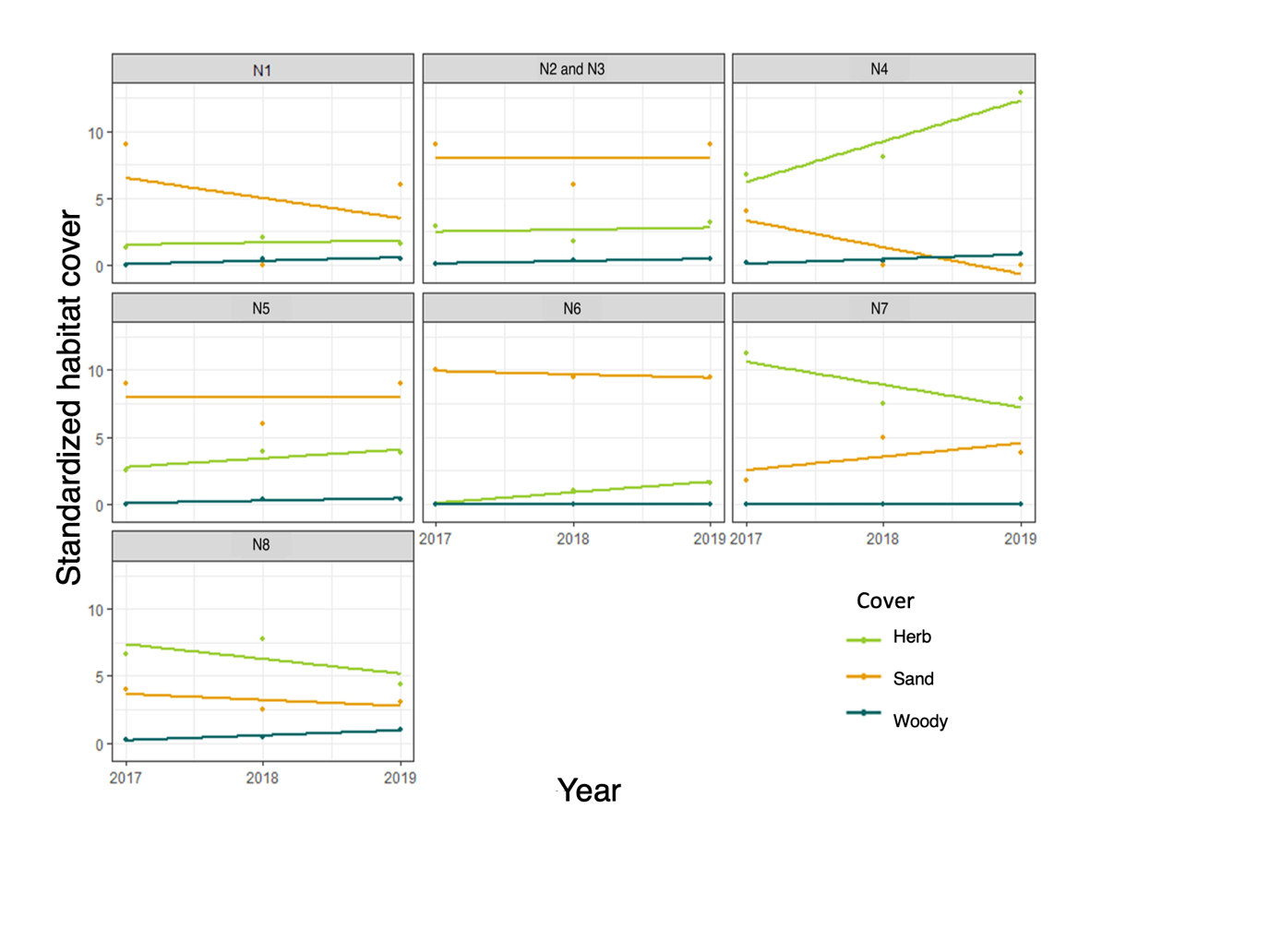


Figure S3: Change in habitat cover across all nesting sites during the sampling years of 2017, 2018 and 2019. We observed a consistent growth in the cover of grass and woody plants, with more frequent decline in sand cover.


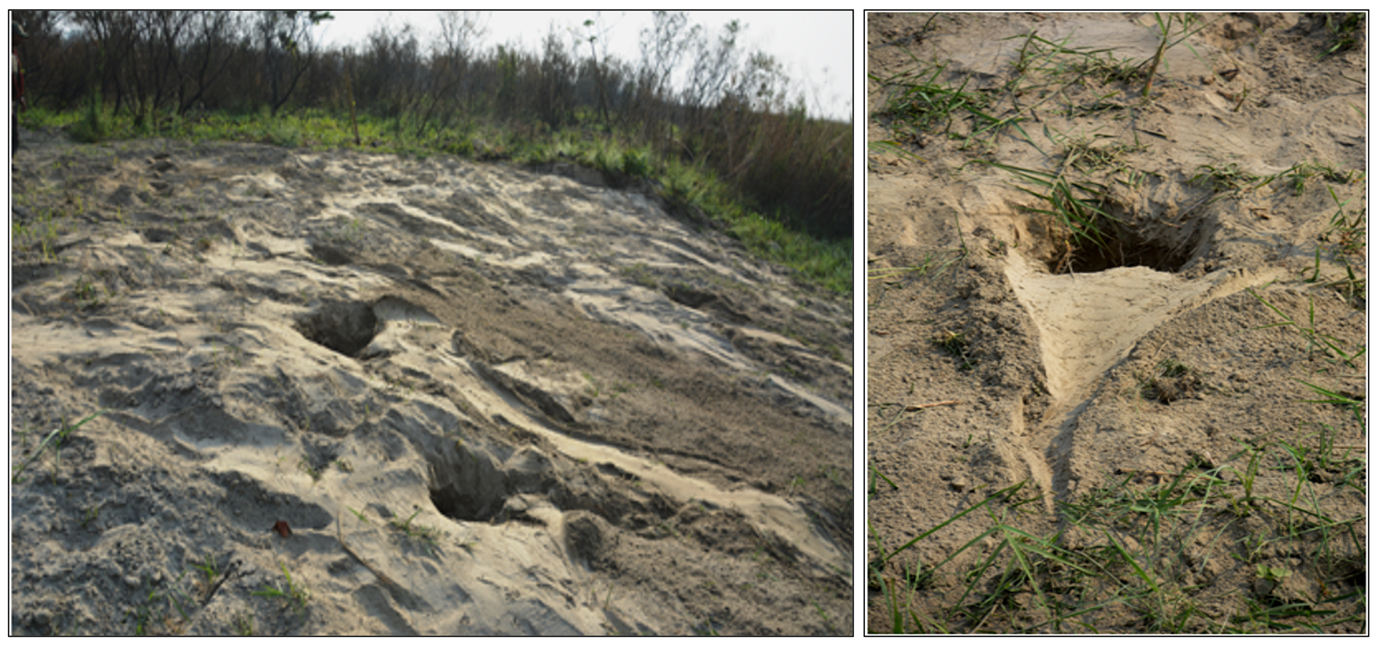


Figure S4: Indirect evidence of gharial nesting around the river in the study area. Trial nests dug by female gharial and spoor marks.
